# The Effect of Ammonia on the Host–Parasite System *Tenebrio molitor* at Different Temperatures

**DOI:** 10.3390/biology15030271

**Published:** 2026-02-03

**Authors:** Denis Rybalka, Viktor Brygadyrenko

**Affiliations:** 1 Department of Biodiversity and Ecology, Oles Honchar Dnipro National University, Nauky Av., 72, 49010 Dnipro, Ukraine; denisrybalka89@gmail.com; 2 Department of Parasitology and Veterinary and Sanitary Expertise, Dnipro State Agrarian and Economic University, Serhii Efremov St., 25, 49000 Dnipro, Ukraine

**Keywords:** *Tenebrio molitor*, *Gregarina cuneata*, *Gregarina polymorpha*, *Gregarina steini*, ammonia, host–parasite interactions, ecological toxicology

## Abstract

Ammonia (NH_3_) is an environmental pollutant that enters ecosystems as a result of agricultural activities, industrial accidents, leaks of ammonia-based rocket fuel, and explosions at chemical plants. Temperature changes can alter the toxicity of ammonia to invertebrates. This study investigated the effect of ammonia on the relationship between *Tenebrio molitor* Linnaeus, 1758 (Coleoptera: Tenebrionidae) and its parasites at temperatures of 21–23 °C and 26–28 °C. We used 150 *T. molitor* larvae, which were divided into five groups of ammonia concentrations (0–4000 mg NH_3_/kg of substrate) at two temperatures (21–23 °C, 26–28 °C). During a 10-day exposure, mortality, body weight changes, and the intensity of parasitic invasion by three species of *Gregarina* were assessed. The results showed a concentration-dependent effect of ammonia on the physiological state and parasitic systems of *T. molitor* (body weight changes: *p* = 2 × 10^−16^; intensity of parasitic invasion: R^2^ = 0.13–0.87), while mortality increased from 0% in the control groups to 40–60% at maximum concentration. Contrary to expectations, temperature did not alter the toxicity of ammonia in the studied range of 21–28 °C (all *p* > 0.18). Parasitological parameters showed higher sensitivity to ammonia stress compared to physiological indicators, forming 4–5 concentration groups versus 2 groups for body weight changes. The observed absence of temperature-dependent changes in ammonia toxicity in the range of 21–28 °C contrasts with the known effects in aquatic invertebrates and may reflect the physiological characteristics of terrestrial insects. The higher sensitivity of parasitological parameters confirms their suitability as indicators of sublethal toxicity for monitoring ammonia pollution in industrial insect breeding systems.

## Simple Summary

This study examined how ammonia pollution affects mealworm larvae (*Tenebrio molitor*) and their gut parasites (*Gregarina* species) at two temperatures. Ammonia enters ecosystems through agricultural activities, industrial accidents, and chemical plant emissions, posing risks to invertebrates. We exposed 150 larvae to different ammonia concentrations (0–4000 mg/kg substrate) at 21–23 °C and 26–28 °C for 10 days, measuring survival, body weight changes, and parasite numbers. Higher ammonia concentrations increased larval mortality (up to 60%) and reduced parasite abundance, but surprisingly, temperature within the tested range did not alter these toxic effects. Parasite counts proved more sensitive to ammonia stress than body weight measurements, with differences detected at lower concentrations. These findings suggest that parasitological parameters could serve as early warning indicators of sublethal ammonia toxicity. The results are relevant for industrial mealworm production, where ammonia accumulates from organic waste decomposition, and for understanding how environmental pollutants affect insect–parasite relationships in agricultural ecosystems.

## 1. Introduction

Ammonia disrupts ecosystem functioning. NH_3_ alters the nitrogen balance and causes cascading effects in food chains [1]. Due to its volatility and solubility, ammonia spreads in the atmosphere and water systems at distances of up to hundreds of kilometers from the source of emission, forming a transboundary impact on remote ecosystems [2]. In aquatic biocenoses, NH_3_ reduces oxygen concentration, causing eutrophication and mass mortality of fish and invertebrates [3]. In terrestrial environments, ammonia contributes to soil acidification and alters plant community composition in agricultural production areas [1]. Agricultural activities generate the bulk of NH_3_ emissions through the use of nitrogen fertilizers and livestock farming. Ammonia evaporation from agricultural land increases during the warm season due to higher temperatures and microbial activity [1]. Industrial ammonia production using the Haber–Bosch process consumes 1–2% of global energy and generates 2.16 kg of CO_2_ per kilogram of product due to raw material conversion and leaks from high-pressure equipment [2,4].

Industrial emissions pose an environmental problem due to sudden releases of concentrated ammonia into the atmosphere, which can spread up to several kilometers from the source [2]. Local sources of pollution include leaks from refrigeration systems, chemical production facilities, and mineral fertilizer storage facilities [2]. The transport and storage of liquid ammonia create risks of emissions in port areas and along railway lines [2]. In aquatic organisms, the toxicity of ammonia shows a temperature dependence [1]. A 5 °C increase in temperature increases the rate of NH_3_ absorption by aquatic organisms by 40% and reduces oxygen solubility, creating a synergistic toxic effect [4].

Global climate change is increasing the toxicity of ammonia in temperate ecosystems [5]. In insects, elevated temperatures reduce symbiont-dependent detoxification metabolism, which affects resistance to toxicants [6]. However, for terrestrial insects, the effect of temperature on ammonia toxicity remains poorly understood, especially in the context of host–parasite interactions. The mealworm beetle *Tenebrio molitor* Linnaeus, 1758 (Coleoptera, Tenebrionidae) is one of the most studied model organisms for toxicological research due to its controlled life cycle and sensitivity to environmental pollutants [7].

*Tenebrio molitor*, as a species, is characterized by controlled growth, high reproductive capacity, and physiological responses to stress factors, characteristics that make it suitable for laboratory experiments [8]. Larvae are used in ecotoxicological studies to assess the effects of heavy metals, pesticides, industrial pollutants, food additives, and surfactants [9,10]. *Tenebrio molitor* allows the study of host–parasite systems under controlled conditions and the assessment of the impact of toxicants on these ecological interactions [11]. The growing demand for alternative protein sources is stimulating the industrial breeding of insects for human consumption and animal feed [12]. *Tenebrio molitor* became the first insect approved by the European Commission as a novel food [13], making it relevant to study the impact of pollutants on the physiological state of organisms and product quality.

In conditions of high-density industrial breeding, the accumulation of organic waste (frass) and its microbial degradation can lead to increased concentrations of ammonia in the substrate and air of production facilities [7], forming a potential stress factor for larvae. Parasitological studies are used to indicate the state of ecosystems, as parasites demonstrate sensitivity to changes in the environment and are an indicator of ecological disturbances [14]. *Gregarina steini*, *G. cuneata*, and *G. polymorpha* are specific parasites of *T. molitor*, whose abundance reflects the host physiological state [15], making them suitable indicators for toxicological studies. Toxic substances affect the intensity of the parasitic load through changes in the host’s physiological responses, disturbances in the intestinal environment, and behavioral changes [16]. Studying the effect of toxicants on parasites allows us to assess the multifactorial impact of pollution at the ecosystem level [17]. Organic xenobiotics do not always alter the load of *T. molitor*; even at elevated concentrations of toxicants, parasites remain viable in the intestinal tract of larvae [11,18]. The predicted increase in temperatures due to global warming [5] highlights the need to study temperature modification of ammonia toxicity for terrestrial invertebrates. Our study aims to assess the effect of ammonia on mortality, body weight, and parasite load in *T. molitor* at two temperatures (21–23 °C and 26–28 °C).

The research hypothesis was that a temperature of 26–28 °C, compared to 21–23 °C, would enhance the toxic effects of ammonia and alter the intensity of *Gregarina* spp. parasite load due to accelerated metabolic processes. The study includes a comparison of physiological and parasitological responses at two temperatures to test for temperature-modified ammonia toxicity in the context of global warming.

## 2. Materials and Methods

**Figure 1:**
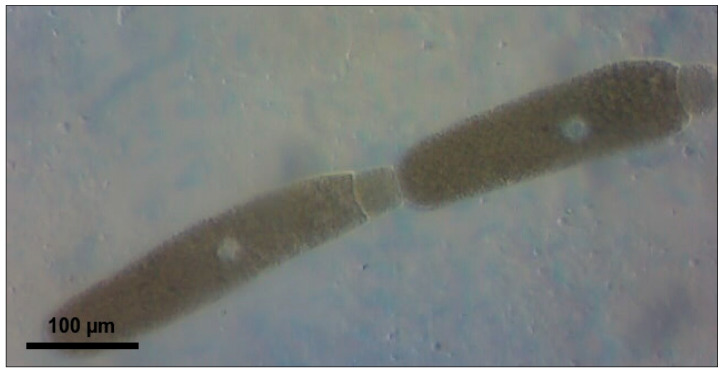
*Gregarina cuneata* syzygy from *Tenebrio molitor* larval midgut; characteristic dumbbell shape with septum constriction between protomerite and deutomerite; nuclei visible as white dots in deutomerites; scale bar = 100 µm.

**Figure 2:**
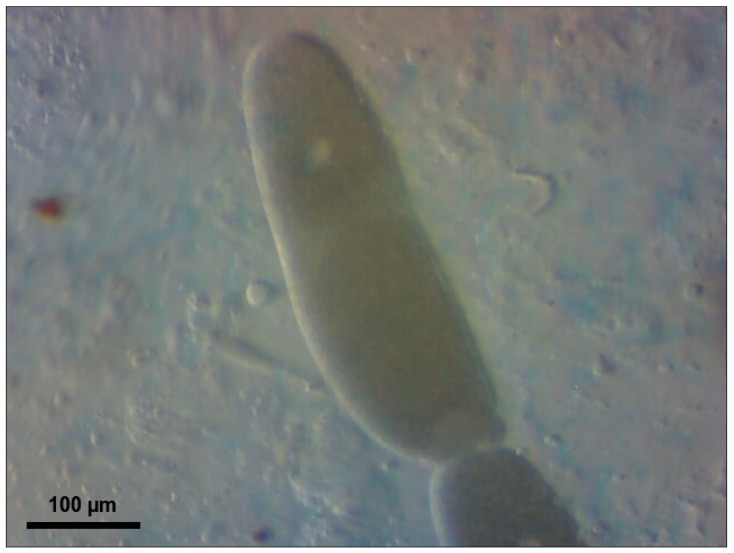
*Gregarina polymorpha* trophozoite from *Tenebrio molitor* larval midgut; elongated cylindrical body without septum constriction; nucleus visible in anterior deutomerite; scale bar = 100 µm.

**Figure 3:**
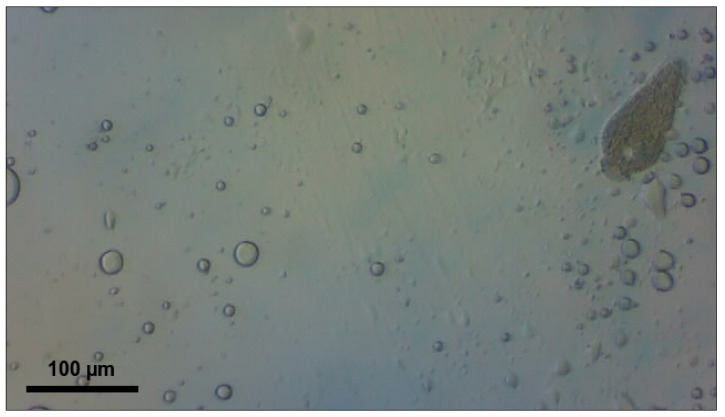
*Gregarina steini* trophozoite from *Tenebrio molitor* larval midgut; small pear-shaped body typical of this species; scale bar = 100 µm.

Larvae from a laboratory culture maintained over a period of 12 months underwent 14-day acclimatization at their respective target temperatures (21–23 °C or 26–28 °C), 45 ± 5% humidity, and a 12:12 photoperiod. The 12:12 photoperiod was selected as a standard laboratory condition, as photoperiod has minimal influence on *T. molitor* development compared to temperature [19]. The diet consisted of pressed oat flakes (LLC ‘Skvyra Bakery Plant’, Skvyra, Ukraine, 2025) produced without pesticide application and cabbage leaves [7,11]. All larvae had equal access to food and equal chances of being infected by parasites. The experimental design included a comparative study of the effect of temperature on ammonia toxicity at two temperatures: 21–23 °C and 26–28 °C. A calibration sieve ensured the selection of 150 larvae with an individual weight of 150 ± 20 mg/specimen. The larvae were randomly assigned to two independent temperature cohorts of 75 individuals each, with each cohort further divided into 5 experimental groups with three replicates of five specimens in each container: the control group (0 mg/kg of feed substrate) and four concentrations of 10% ammonia solution (1000, 2000, 3000, and 4000 mg NH_3_/kg). Two temperature experiments were conducted consecutively with a 30-day interval between stages to exclude seasonal effects on the physiological state of the larvae and to ensure identical laboratory conditions. The exposure lasted 10 days under a 12:12 photoperiod and 45 ± 5% humidity to ensure reproducibility of the results. Complete individual data for each larva are provided in Supplementary Table S1 (temperature 21–23 °C, *n* = 75) and Supplementary Table S2 (temperature 26–28 °C, *n* = 75), including initial and final body weights (mg/specimen), changes in body weight (mg/specimen), and the number of parasites of three *Gregarina* species (specimens/larva) for each concentration group. A 10% aqueous solution of ammonia (100 mg NH_3_/mL) was stored at 4 °C in sealed glass bottles for no more than 7 days to prevent degradation. Concentrations are reported as total ammonia equivalents (mg NH_3_/kg substrate) based on nominal solution concentrations. The calculated volumes of the solution (1.00–4.00 mL, corresponding to 1000–4000 mg NH_3_/kg of substrate) were added with a micropipette to 100 ± 5 g of pressed oat grains at a depth of 50 mm from the surface to ensure uniform distribution in the substrate. To control for moisture effects, all groups received equal total liquid volumes (4.00 mL per 100 g substrate). When ammonia solution volumes were below 4.00 mL, the difference was supplemented with distilled water (e.g., 1.00 mL ammonia + 3.00 mL water for 1000 mg/kg). Control groups received 4.00 mL of distilled water only. Transparent plastic containers (500 mL) with snap-on lids were equipped with a cotton swab to absorb moisture and 0.5 g/container of cabbage leaves, pre-cut into 2 × 2 cm squares. Larvae (5 per container, 3 replicates per concentration) were introduced after ammonia application to avoid direct contact with concentrated solution. The containers were sealed with 2 mm diameter ventilation holes to maintain aerobic conditions while minimizing ammonia volatilization. Atmospheric ammonia was monitored using an MQ-137 sensor (Zhengzhou Winsen Electronics Technology Co., Ltd., Zhengzhou, China, 2024) connected to ESP32 microcontroller (Espressif Systems, Shanghai, China, 2024), with measurements recorded at 1 min intervals. Atmospheric concentrations declined below the sensor detection limit within the initial hours due to volatilization; however, larvae remained in continuous contact with ammonia-treated substrate throughout the 10-day exposure period. After 10 days of exposure, each larva was weighed individually on an analytical scale with an accuracy of ±1 mg/specimen, and a parasitological examination of the intestine was performed. The midgut (from the stomach to the Malpighian vessels) was removed with a sterile scalpel and placed in a drop of physiological solution (0.9% NaCl) on a microscope slide to prevent drying. Transverse incisions (12 incisions at 0.5 mm intervals) ensured complete release of intestinal contents, with attention paid to the posterior part of the midgut, where *Gregarina* spp. trophozoites are located [15]. Within three minutes of dissection, the larval midgut was systematically scanned under a phase-contrast light microscope at ×400 and ×1000 magnification. Only freely motile trophozoites released from the midgut during standardized compression with a coverslip were counted; immature gamonts attached to the gut wall via the epimerite were excluded. All dissections and counts were performed by a single trained observer (DFR) to ensure internal consistency. We acknowledge that a small proportion of trophozoites may have remained inside the gut; however, consistency was maintained across all samples by applying the same dissection protocol, including standardized incisions, equal volumes of 0.9% NaCl solution, and uniform pressure applied via coverslip. *G. cuneata* (Figure 1), *G. polymorpha* (Figure 2), and *G. steini* (Figure 3) were identified through morphometric measurements according to Clopton & Janovy [14]. The total number of parasites of each species (specimens/larva) was counted.

Homogeneity of variances was tested using the Levene test with grouping by temperature and concentration. Under these conditions, the F-test maintained robust Type I error rates even with non-normal distributions [20]. The normality of the distribution of the residuals of the two-factor ANOVA model was assessed using the Shapiro–Wilk test (W = 0.85, *p* = 8.3 × 10^−10^), which revealed deviations from normal distribution (Supplementary Figure S1 shows the Q–Q plot of ANOVA residuals). Since the homogeneity of variances was satisfied (*p* = 0.35), and since variance analysis is robust to moderate violations of normality under balanced experimental designs and homogeneous variances [20], a two-way ANOVA was used to test the effect of temperature, concentration, and their interaction on daily body weight changes. The statistical significance of the main effects and interactions was assessed using F-statistics with the corresponding degrees of freedom. To determine statistical differences between concentration groups, we used Tukey’s post hoc test in the emmeans package [21], with correction for multiple comparisons. The results were visualized using a compact letter display, where groups with the same letters have no statistically significant differences at a level of *p* = 0.05 [22]. Additionally, linear regression analysis was performed to quantitatively assess the relationship between ammonia concentration (as a continuous numerical variable) and changes in body weight separately for each temperature group, with the calculation of coefficients of determination (R^2^) to estimate the proportion of explained variance. Mortality was modeled using binomial generalized linear models (GLMs) with the logit link function, with concentration (numeric) and temperature as fixed effects. Model comparison was performed using likelihood ratio tests. The number of parasites (*G. cuneata*, *G. polymorpha*, and *G. steini*) was analyzed using generalized linear models (GLMs) with a negative binomial distribution using the MASS package [23], as the data showed overdispersion typical of parasite count data and contained 31–35% of zero values, which is typical for parasite counts in ecological studies [24,25,26]. The negative binomial distribution naturally accommodates zero counts within its probability mass function [27]. Combined GLM models with concentration, temperature, and their interaction were fitted to test for temperature effects; concentration effects were additionally assessed within each temperature regime. For each parasite species, a model with concentration as a continuous variable was selected to assess the concentration effect separately for each temperature group. The statistical significance of the concentration effect was assessed using likelihood ratio tests by comparing models with concentration and a baseline model without predictors. To determine statistical differences between concentration groups, we used Tukey’s post hoc test in the emmeans package [21]. Coefficients of determination (pseudo-R^2^) were calculated as 1—(deviance_model/deviance_null), reflecting the proportion of variation in parasite numbers explained by ammonia concentration. The total parasite load (sum of all three *Gregarina* species) was analyzed similarly using a GLM with a negative binomial distribution to assess the cumulative effect of ammonia stress on parasite communities. The model included concentration as a continuous predictor with separate analyses for each temperature group. Post hoc comparisons between concentration groups were performed similarly. Post hoc power analysis indicated that with N = 150 and α = 0.05, the study had 80% power to detect effect sizes of Cohen’s w ≥ 0.23, which was sufficient for the observed concentration (w = 0.44) and temperature (w = 0.22) effects. Statistical analyses were performed in R version 4.4.2 [28] in RStudio version 2024.12.0–467 using the following packages: dplyr (version 1.1.4) for data processing, ggplot2 (version 3.5.1) for visualisation, MASS (version 7.3-61) for negative binomial regression, car (version 3.1-3) for Levene’s tests and analysis of variance, lmtest (version 2.0-40) for likelihood ratio tests, emmeans (version 1.10.0) for estimating marginal means, and multcomp (version 1.4-26) for compact letter mapping.

## 3. Results

### 3.1. Mortality and Body Weight of T. molitor

**Figure 4:**
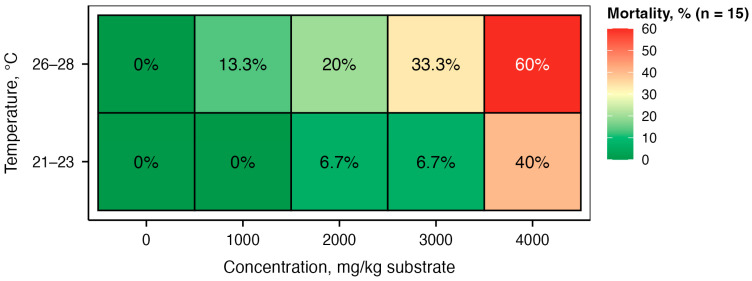
Mortality of *Tenebrio molitor* larvae after 10 days of exposure to different concentrations of ammonia in the feed substrate at two temperatures: the heatmap shows the percentage of mortality (numerical values in cells) with a color gradient from green (0% mortality) to red (60% mortality).

Larval mortality increased with increasing ammonia concentration (Figure 4). Under control conditions (0 mg NH_3_/kg substrate), mortality was 0% for both temperatures and increased to 6.7% and 33.3% at 3000 mg NH_3_/kg substrate and to 40% and 60% at 4000 mg NH_3_/kg substrate for 21–23 °C and 26–28 °C, respectively. Binomial GLM analysis revealed significant main effects of both ammonia concentration (χ^2^ = 29.56, *p* < 0.001) and temperature (χ^2^ = 7.00, *p* = 0.008) on larval survival. The concentration × temperature interaction was not significant (χ^2^ = 1.04, *p* = 0.308), indicating that the dose–response relationship was consistent across both temperature regimes.

**Figure 5:**
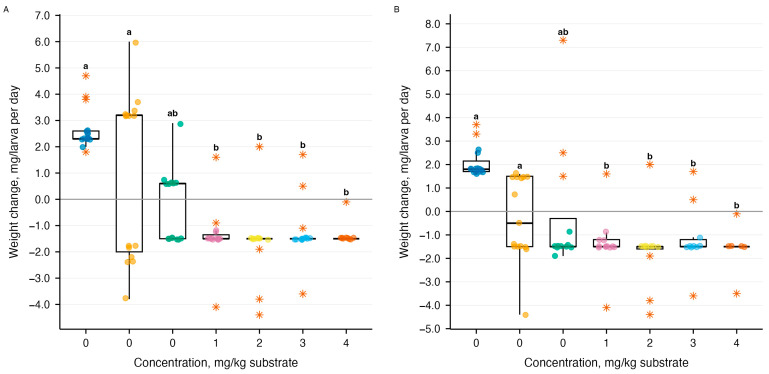
Effect of ammonia on daily changes in body weight of *Tenebrio molitor* larvae during a 10-day experiment: (**A**) temperature 21–23 °C (*n* = 63), (**B**) temperature 26–28 °C (*n* = 60); boxplots show the distribution of body weight changes; the thick black line inside the box is the median, the box boundaries are the first and third quartiles (Q1, Q3); the whiskers of the boxplots are the range of typical values within 1.5 × IQR from the box boundaries; colored circles—individual measurements for each larva; orange stars (*)—statistical outliers; letters above boxplots—results of Tukey’s post hoc test (groups with the same letters have no statistically significant differences at *p* = 0.05).

The Levene test confirmed homogeneity of variances (F_9,140_ = 1.13, *p* = 0.35). Among the larvae that survived the experiment (*n* = 123), two-factor analysis of variance revealed a statistically significant effect of ammonia concentration on daily body weight changes (F_4,113_ = 52.64, *p* = 2 × 10^−16^), while the effect of temperature (F_1,113_ = 0.01, *p* = 0.92) and the interaction between temperature and concentration (F_4,113_ = 0.48, *p* = 0.75) were statistically insignificant. Ammonia concentration explained 68.4% of the variance at temperatures of 21–23 °C (R^2^ = 0.68, *p* = 1.28 × 10^−13^) and 61.2% at temperatures of 26–28 °C (R^2^ = 0.61, *p* = 3.35 × 10^−9^) (Figure 5). Tukey’s post hoc test showed that the control group (0 mg NH_3_/kg substrate) differed significantly from all experimental groups (*p* < 0.0001), demonstrating a weight gain of +2.38 ± 0.23 mg/larva per day. Starting from a concentration of 1000 mg/kg, a weight loss of −1.19 to −1.69 mg/larva per day was observed, with no statistically significant differences between concentrations of 1000–4000 mg/kg (all pairwise comparisons *p* > 0.75).

### 3.2. Invasion of Gregarina spp. in T. molitor

**Figure 6:**
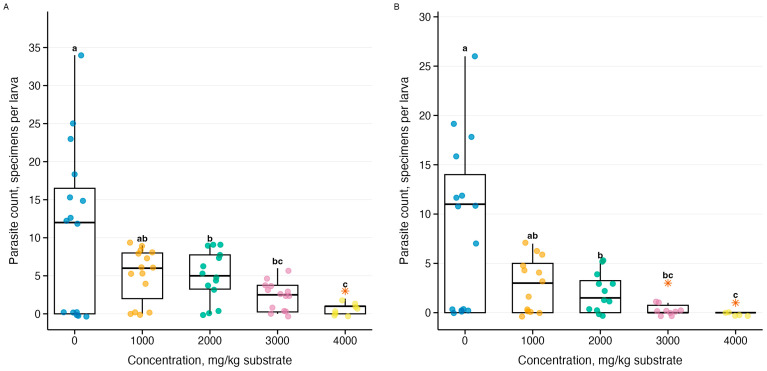
Effect of ammonia on the intensity of *Gregarina cuneata* parasitic invasion in *Tenebrio molitor* larvae during a 10-day experiment: (**A**) temperature 21–23 °C (*n* = 63), (**B**) temperature 26–28 °C (*n* = 60); see Figure 5; orange stars (*)—statistical outliers; letters above boxplots—results of Tukey’s post hoc test averaged for both temperatures.

**Figure 7:**
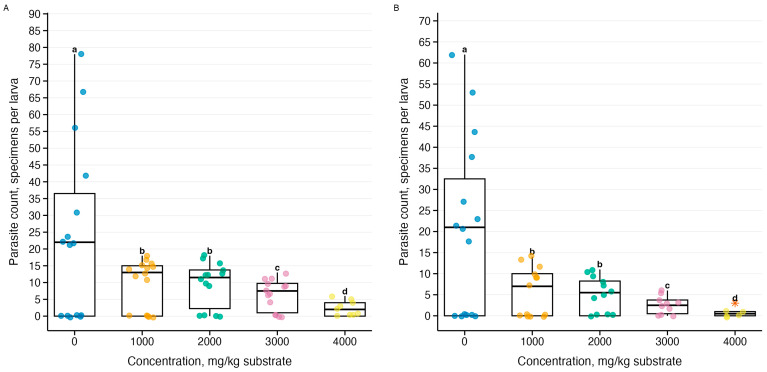
Effect of ammonia on the intensity of *Gregarina polymorpha* parasitic invasion in *Tenebrio molitor* larvae during a 10-day experiment: (**A**) temperature 21–23 °C (*n* = 63), (**B**) temperature 26–28 °C (*n* = 60); see explanation in Figure 5; orange stars (*)—statistical outliers; letters above boxplots—results of Tukey’s post hoc test averaged for both temperatures.

**Figure 8:**
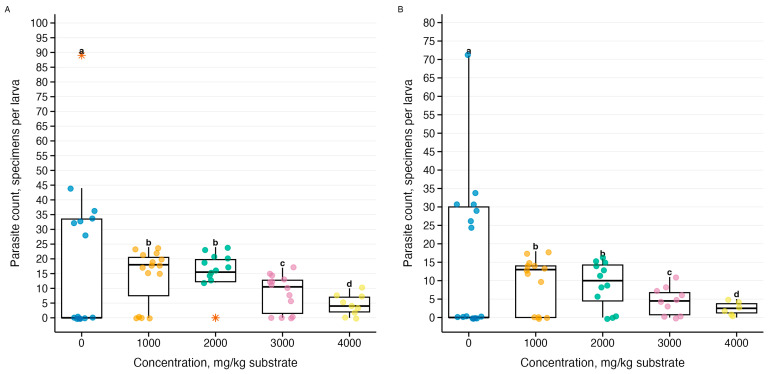
Effect of ammonia on the intensity of *Gregarina steini* parasitic invasion in *Tenebrio molitor* larvae during a 10-day experiment: (**A**) temperature 21–23 °C (*n* = 63), (**B**) temperature 26–28 °C (*n* = 60); see Figure 5; orange stars (*)—statistical outliers; letters above boxplots—results of Tukey’s post hoc test averaged for both temperatures.

Parasite count data showed overdispersion (variance-to-mean ratio = 6.5–15.7), justifying the use of negative binomial GLM. Analysis of the effect of ammonia on the intensity of parasitic invasion revealed species-specific responses of three *Gregarina* species (Figure 6, Figure 7 and Figure 8). Generalized linear models with negative binomial distribution showed varying explanatory power: the strongest response was demonstrated by *G. cuneata* (R^2^ = 0.84–0.87, *p* = 3.27 × 10^−78^–3.44 × 10^−89^), intermediate sensitivity was demonstrated by *G. polymorpha* (R^2^ = 0.26–0.36, *p* = 2.76 × 10^−62^–1.48 × 10^−71^), and the weakest dependence was demonstrated by *G. steini* (R^2^ = 0.13–0.17, *p* = 9.74 × 10^−30^–9.78 × 10^−31^). The intensity of parasitic invasion decreased from control conditions to the maximum concentration for all three species. Post hoc analysis revealed statistically significant decreases from the lowest concentration of 1000 mg NH_3_/kg substrate.

**Figure 9:**
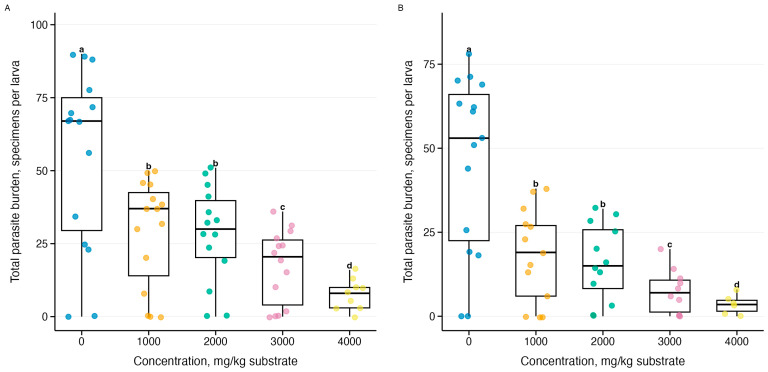
Effect of ammonia on the total parasite load (sum of all three *Gregarina species*) in *Tenebrio molitor* larvae during a 10-day experiment: (**A**) temperature 21–23 °C (*n* = 63), (**B**) temperature 26–28 °C (*n* = 60); see Figure 5; letters above boxplots—results of Tukey’s post hoc test averaged for both temperatures.

Analysis of total parasitic infestation (sum of all three *Gregarina* species) revealed a dose-dependent effect of ammonia stress (Figure 9). Generalized linear models with negative binomial distribution explained 38–47% of the variance (R^2^ = 0.38–0.47, *p* = 4.98 × 10^−103^–1.31 × 10^−99^ at both temperatures), demonstrating a decrease in infestation from control conditions to a maximum concentration of 4000 mg/kg.

### 3.3. Temperature Effects on the T. molitor–Gregarina System

Analysis of the temperature effect did not reveal any statistically significant effects on any of the parameters studied. Two-factor ANOVA for body weight changes did not reveal any effect of temperature (F_1,113_ = 0.01, *p* = 0.92) or temperature × concentration interaction (F_4,113_ = 0.48, *p* = 0.75). Generalized linear models did not reveal a temperature effect on the intensity of invasion by *G. cuneata* (χ^2^ = 1.79, df = 1, *p* = 0.18), *G. polymorpha* (χ^2^ = 0.84, df = 1, *p* = 0.36) or *G. steini* (χ^2^ = 1.04, df = 1, *p* = 0.31).

## 4. Discussion

### 4.1. Ammonia Toxicity to T. molitor

The present study demonstrated a clear concentration-dependent increase in mortality of *T. molitor* individuals, with mortality rising to 60% at 4000 mg NH_3_/kg of substrate, which is consistent with the known toxic effects of ammonia reported for aquatic invertebrates [1,29]. The non-ionized form of NH_3_ easily penetrates cell membranes, disrupting osmoregulation and ion balance [1]. A similar concentration-dependent mortality was observed in *Aedes triseriatus* mosquito larvae [29].

### 4.2. Gregarina spp. as Indicators of Toxic Stress

The effect of ammonia on the host–parasite system manifests itself at several levels: toxic stress can simultaneously affect *T. molitor* and directly affect *Gregarina* spp. The decrease in the intensity of invasion of all three *Gregarina* species with increasing ammonia concentration is consistent with the concept of combined effects of stressors on host-parasite systems [30,31,32]. Most studies have been conducted on aquatic systems, and our study extends this concept to terrestrial invertebrates. Gregarines have adaptations to the intestinal environment of invertebrates, including specific attachment and feeding mechanisms [14]. Their tolerance to chemical stressors may differ from that of their host. Studies by Clopton et al. [14,33] have shown that different species of *Gregarina* in *T. molitor* have specific localization and sensitivity to changes in the physiological state of the host, which may explain species-specific differences in response to ammonia stress. Ammonia can affect host–parasite systems through two pathways. First, ammonia disrupts host midgut physiology, wherein elevated ammonia alters intestinal pH and acid–base balance [34], which directly affects *Gregarina* since their excystation and attachment depend on midgut pH conditions [35]. Second, ammonia-induced stress reduces host nutritional status, and since gregarines consume nutrients from the host midgut [18], nutritionally compromised hosts support lower parasite loads. Under stress conditions, the parasitic load has a greater negative effect on the host [15]. Individuals with a high parasitic load die first, so the intensity of invasion is lower among those that survive. At 21–23 °C with 1000 mg NH_3_/kg substrate, all larvae survived yet parasite intensity was reduced, indicating that selective mortality of heavily-infected individuals cannot fully explain the observed parasite reduction. At higher concentrations where mortality was substantial, selective mortality may contribute to the effect, representing a limitation of the current study design.

### 4.3. Temperature Effects

The initial hypothesis of the study about the temperature-induced increase in ammonia toxicity was not confirmed by the experimental results. Unlike the temperature effects on aquatic species described in the literature, our data revealed no effect of temperature on all studied parameters in the range of 21–28 °C (all *p* > 0.18). Previous studies on aquatic invertebrates have demonstrated an increase in ammonia toxicity at temperatures above 25 °C due to accelerated metabolism and biochemical processes [1,4]. The absence of a temperature effect can be explained by several factors. First, *T. molitor* larvae, as terrestrial insects, have thermoregulatory mechanisms that differ from those of aquatic species. A study by Carter et al. [36] showed that terrestrial ectotherms increase their standard metabolic rate with temperature variability (+2.8% per °C), while aquatic organisms decrease it (−6.8% per °C). Secondly, ammonia in the substrate environment has different volatility and availability dynamics than in an aqueous solution, where temperature affects the concentration of dissolved NH_3_. The use of sealed containers with lids minimized ammonia volatilization loss, ensuring that larvae were exposed to substrate-derived ammonia throughout the experimental period. The narrow temperature range tested (21–28 °C) may have been insufficient to reveal temperature-dependent effects; however, this range represents ecologically relevant conditions for *T. molitor* in industrial production and temperate agricultural settings. The sequential execution of temperature treatments may introduce batch effects, though each cohort included internal controls and concentration–response patterns remained consistent across regimes. Future studies should examine wider thermal gradients to determine threshold temperatures for ammonia toxicity modulation.

### 4.4. Parasitic Load as an Indicator of Toxicity

The intensity of parasitic invasion formed four statistically distinct concentration groups according to Tukey’s test, while body weight formed only two. The coefficients of determination for the parasite load models were R^2^ = 0.38 (21–23 °C) and R^2^ = 0.47 (26–28 °C). The differential sensitivity of parasites and hosts to pollutants has been documented in ecotoxicological studies, where parasites act as indicators of stressors at different levels of biological organization [31,32,37]. Individual *Gregarina* species also showed different sensitivities: *G. cuneata* showed R^2^ = 0.84–0.87, *G. polymorpha*—R^2^ = 0.26–0.36, while *G. steini*—R^2^ = 0.13–0.17.

### 4.5. Limitations of the Study

The duration of exposure (10 days) allowed us to identify acute effects on mortality, body weight, and parasite load, but it did not cover the chronic effects of ammonia on the entire life cycle of *T. molitor* from the larval to the adult stage. Research by Deruytter et al. [38] demonstrated that physiological changes in larvae may manifest after a longer observation period. The lack of assessment of late effects (delayed toxicity) and effects on reproductive potential limits conclusions about the population consequences of ammonia exposure. Baseline parasite loads could not be ascertained for individual larvae because parasitological examination requires lethal dissection of the midgut to count trophozoites; however, previous studies from our laboratory using the same *T. molitor* culture have established consistent baseline parasite loads under control conditions (Rybalka & Brygadyrenko, 2025; Lazurska & Brygadyrenko, 2024) [11,18], providing confidence that random assignment from this homogeneous population produces comparable initial infection levels across treatment groups. The temperature range of 21–28 °C is within the optimum range for *T. molitor* [19], which may explain the absence of temperature effects in our study. Extending the temperature range to extreme values (10–15 °C or 35–40 °C) could reveal temperature-dependent modifications of ammonia toxicity. Using only two temperature regimes does not allow for the detection of nonlinear temperature dependencies or threshold effects. The experimental model was based on a single type of substrate (wheat bran), whereas in natural conditions, *T. molitor* larvae can consume a variety of organic materials with different nitrogen and moisture contents, which affects their physiological response [39]. Laboratory conditions with controlled humidity and no natural predators may not reflect the complexity of field populations. The study focused on individual indicators (body weight, mortality) and parasite load without assessing behavioral changes, which may be sensitive indicators of sublethal toxicity [40]. The lack of analysis of biochemical indicators (detoxification enzyme activity and ammonia metabolite concentration) limits our understanding of the mechanisms of toxic action at the cellular and molecular levels. The interaction of ammonia with other stressors (heavy metals, pesticides, and pathogens) was not assessed, while synergistic or antagonistic effects of multiple stressors are typical for polluted agroecosystems [37]. The lack of comparison with other host insect species limits the possibility of extrapolating the results to broader ecological contexts.

### 4.6. Significance for Edible Insect Production

The growing use of *T. molitor* as a source of protein for human consumption and animal feed [39] makes it important to control parasitic infections and chemical stressors in mass rearing [41]. The IoT monitoring system using MQ-137 sensor shows potential as a cost-effective tool for environmental assessment in *T. molitor* industrial production, where ammonia control is important for product quality [39,41]. However, field validation studies under commercial production conditions are needed to confirm the applicability of laboratory-derived thresholds.

## 5. Conclusions

The study revealed a concentration-dependent effect of ammonia on *Tenebrio molitor* larvae and their parasitic systems in the absence of temperature-modified toxicity in the range of 21–28 °C. Contrary to the initial hypothesis, temperature did not modify the toxicity of ammonia for any of the parameters studied (all *p* > 0.18), which contrasts with the known effects in aquatic invertebrates and may reflect the physiological characteristics of terrestrial insects. A key finding is the higher sensitivity of parasitological parameters to ammonia stress compared to host physiological parameters. The intensity of parasitic invasion formed four statistically distinct concentration groups according to Tukey’s test, while changes in body mass showed only a binary response. Three species of *Gregarina* showed species-specific sensitivity: *G. cuneata* (R^2^ = 0.84–0.87), *G. polymorpha* (R^2^ = 0.26–0.36), and *G. steini* (R^2^ = 0.13–0.17). The study extends the concept of using parasites as indicators of ecological stress from aquatic to terrestrial invertebrate systems. The combination of IoT monitoring with biological indicator systems shows potential for environmental assessment in ecotoxicological studies. However, field validation is recommended before the application of *T. molitor* in industrial production settings. Parasitological parameters can serve as sensitive bioindicators for the early detection of ammonia stress in monitoring protocols. Further studies should include chronic exposures throughout the entire life cycle, extension of the temperature range beyond optimal conditions (15–18 °C and 30–33 °C), assessment of behavioral and biochemical indicators of sublethal toxicity, and field verification under multiple stressors.

## Data Availability

Data supporting this study are openly available at hosted on GitHub: https://github.com/RybalkaDenis/ammonia_on_host_parasite_system (accessed on 10 December 2025).

## Supplementary Materials

The following supporting information can be downloaded at: https://www.mdpi.com/article/10.3390/biology15030271/s1, Figure S1: Q–Q plot of ANOVA residuals for body weight changes; Table S1: Individual experimental data for 75 *Tenebrio molitor* larvae (body weight, *Gregarina* spp. parasite counts, and mortality status) during 10-day ammonia exposure (0–4000 mg NH_3_/kg substrate) at 21–23 °C; Table S2: Individual experimental data for 75 *Tenebrio molitor* larvae (body weight, *Gregarina* spp. parasite counts, and mortality status) during 10-day ammonia exposure (0–4000 mg NH_3_/kg substrate) at 26–28 °C.
