# Supplementary material for: The Effect of Ammonia on the Host–Parasite System Tenebrio molitor at Different Temperatures
Source: Biology (Basel). 2026 Feb 3;15(3):271. doi: 10.3390/biology15030271 (PMC12896862; doi:10.3390/biology15030271)
Supplement: Supplementary file 1 [file biology-15-00271-s001.zip › biology-4072353-supplementary.pdf]

Table S1. Complete experimental data for temperatures of 21–23 °C. Individual experimental data for all *Tenebrio molitor* larvae at temperatures of 21–23 °C during 10 days of exposure to different concentrations of 10% ammonium solution. Columns: ID—unique larval identifier; Temperature—temperature regime (°C); 10% NH_3—ammonium concentration (mg/100g substrate); Start/End—body weight (mg); Δ—weight change (mg); G.p., G.c., and G.s.—*Gregarina polymorpha, G. cuneata,* and *G. steini* counts (specimens/larva), respectively; Status—survival outcome.

Temperature, °C	10% NH_3	Start weight, mg	End weight, mg	Weight change, mg	*G.p.*	*G.c.*	*G.s.*	Status
21–23	0	160	207	47	42	18	28	Alive
21–23	0	161	200	39	0	0	0	Alive
21–23	0	137	155	18	67	0	0	Alive
21–23	0	156	176	20	24	12	36	Alive
21–23	0	154	192	38	0	25	0	Alive
21–23	0	142	168	26	22	13	32	Alive
21–23	0	148	171	23	0	0	89	Alive
21–23	0	163	186	23	31	15	44	Alive
21–23	0	139	164	25	0	0	0	Alive
21–23	0	155	178	23	0	23	0	Alive
21–23	0	146	172	26	56	0	0	Alive
21–23	0	158	181	23	22	12	33	Alive
21–23	0	151	174	23	0	34	0	Alive
21–23	0	144	169	25	21	15	34	Alive
21–23	0	162	185	23	78	0	0	Alive
21–23	1000	166	182	16	14	7	19	Alive
21–23	1000	131	119	−12	0	0	0	Alive
21–23	1000	180	168	−12	13	6	18	Alive
21–23	1000	196	155	−41	15	9	22	Alive
21–23	1000	126	117	−9	0	8	0	Alive
21–23	1000	173	158	−17	18	8	23	Alive
21–23	1000	145	130	−19	12	5	15	Alive
21–23	1000	189	174	−15	0	0	0	Alive
21–23	1000	157	142	−13	14	6	17	Alive
21–23	1000	164	149	−18	16	8	21	Alive
21–23	1000	178	163	−16	0	0	20	Alive
21–23	1000	152	137	−17	15	5	18	Alive
21–23	1000	161	146	−12	11	4	15	Alive
21–23	1000	148	133	−15	17	9	24	Alive
21–23	1000	182	167	−20	0	0	0	Alive
21–23	2000	185	141	−44	12	5	15	Alive
21–23	2000	216	197	−19	0	0	0	Alive
21–23	2000	154	174	20	16	8	21	Alive
21–23	2000	158	178	20	13	6	17	Alive
21–23	2000	184	146	−38	0	0	19	Alive
21–23	2000	167	152	−18	11	4	13	Alive
21–23	2000	149	134	−16	17	9	23	Alive
21–23	2000	173	158	−12	9	3	12	Alive
21–23	2000	191	176	−9	0	0	0	Alive
21–23	2000	156	141	−12	12	5	16	Alive
21–23	2000	178	163	−19	14	7	20	Alive
21–23	2000	162	147	−22	10	4	14	Alive

21–23	2000	145	0	0	0	0	0	Dead
21–23	2000	187	172	−8	0	9	0	Alive
21–23	2000	174	159	−14	18	9	24	Alive
21–23	3000	164	153	−11	9	3	12	Alive
21–23	3000	162	126	−36	11	5	15	Alive
21–23	3000	154	139	−11	0	0	0	Alive
21–23	3000	134	139	5	13	6	17	Alive
21–23	3000	132	149	17	10	4	13	Alive
21–23	3000	178	163	−13	6	1	8	Alive
21–23	3000	156	141	−13	0	0	0	Alive
21–23	3000	149	0	0	0	0	0	Dead
21–23	3000	167	152	−12	8	3	11	Alive
21–23	3000	143	128	−16	0	2	0	Alive
21–23	3000	171	156	−16	11	4	14	Alive
21–23	3000	159	144	−17	9	3	12	Alive
21–23	3000	184	169	−19	4	0	6	Alive
21–23	3000	148	133	−21	0	0	0	Alive
21–23	3000	161	146	−21	7	2	10	Alive
21–23	4000	149	134	−18	5	1	7	Alive
21–23	4000	164	0	0	0	0	0	Dead
21–23	4000	149	0	0	0	0	0	Dead
21–23	4000	159	144	−20	0	0	0	Alive
21–23	4000	142	141	−1	6	2	8	Alive
21–23	4000	176	161	−16	2	0	3	Alive
21–23	4000	158	143	−23	0	0	10	Alive
21–23	4000	173	0	0	0	0	0	Dead
21–23	4000	151	136	−13	4	1	5	Alive
21–23	4000	167	0	0	0	0	0	Dead
21–23	4000	145	0	0	0	0	0	Dead
21–23	4000	182	167	−11	1	0	2	Alive
21–23	4000	154	0	0	0	0	0	Dead
21–23	4000	168	153	−12	0	3	0	Alive
21–23	4000	179	164	−20	3	1	4	Alive

Table S2. Complete experimental data for temperatures of 26–28 °C**.** Individual experimental data for all *Tenebrio molitor* larvae at temperatures of 26–28 °C during 10 days of exposure to different concentrations of 10% ammonium solution. Column descriptions as in Table S1.

**Temperature, °C**	**10% NH_3**	**Start weight, mg**	**End weight, mg**	**Weight change, mg**	***G.p.***	***G.c.***	***G.s.***	**Status**
26–28	0	158	195	37	38	16	24	Alive
26–28	0	162	188	26	0	0	0	Alive
26–28	0	144	162	18	53	0	0	Alive
26–28	0	157	174	17	21	11	31	Alive
26–28	0	149	182	33	0	19	0	Alive
26–28	0	153	169	16	18	7	26	Alive
26–28	0	166	191	25	0	0	71	Alive
26–28	0	140	158	18	27	12	31	Alive
26–28	0	161	179	18	0	0	0	Alive
26–28	0	145	163	18	0	18	0	Alive

26–28	0	159	176	17	44	0	0	Alive
26–28	0	152	170	18	21	11	29	Alive
26–28	0	148	166	18	0	26	0	Alive
26–28	0	164	181	17	23	12	34	Alive
26–28	0	156	173	17	62	0	0	Alive
26–28	1000	166	182	16	10	4	13	Alive
26–28	1000	131	119	−12	0	0	0	Alive
26–28	1000	180	168	−12	0	3	12	Alive
26–28	1000	196	155	−41	9	0	14	Alive
26–28	1000	126	117	−9	0	6	0	Alive
26–28	1000	173	158	−17	13	7	17	Alive
26–28	1000	145	0	0	0	0	0	Dead
26–28	1000	189	174	−19	0	0	0	Alive
26–28	1000	157	142	−15	14	6	18	Alive
26–28	1000	164	0	0	0	0	0	Dead
26–28	1000	178	163	−13	0	0	13	Alive
26–28	1000	152	137	−18	12	5	15	Alive
26–28	1000	161	146	−16	7	2	10	Alive
26–28	1000	148	133	−17	9	4	14	Alive
26–28	1000	182	167	−12	0	0	0	Alive
26–28	2000	185	141	−44	6	1	9	Alive
26–28	2000	216	197	−19	0	0	0	Alive
26–28	2000	154	174	20	10	5	15	Alive
26–28	2000	158	178	20	7	2	11	Alive
26–28	2000	184	146	−38	0	0	13	Alive
26–28	2000	167	0	0	0	0	0	Dead
26–28	2000	149	134	−18	5	1	8	Alive
26–28	2000	173	158	−16	11	5	16	Alive
26–28	2000	191	0	0	0	0	0	Dead
26–28	2000	156	141	−12	0	0	0	Alive
26–28	2000	178	163	−9	8	3	14	Alive
26–28	2000	162	147	−12	4	0	6	Alive
26–28	2000	145	0	0	0	0	0	Dead
26–28	2000	187	172	−19	0	3	0	Alive
26–28	2000	174	159	−22	9	4	15	Alive
26–28	3000	164	153	−11	3	0	5	Alive
26–28	3000	162	126	−36	5	1	8	Alive
26–28	3000	154	0	0	0	0	0	Dead
26–28	3000	134	139	5	0	0	0	Alive
26–28	3000	132	149	17	6	3	11	Alive
26–28	3000	178	0	0	0	0	0	Dead
26–28	3000	156	141	−11	2	0	4	Alive
26–28	3000	149	0	0	0	0	0	Dead
26–28	3000	167	152	−13	0	0	0	Alive
26–28	3000	143	0	0	0	0	0	Dead
26–28	3000	171	156	−13	3	1	6	Alive
26–28	3000	159	144	−12	4	0	7	Alive
26–28	3000	184	0	0	0	0	0	Dead
26–28	3000	148	133	−16	0	0	0	Alive
26–28	3000	161	146	−16	2	0	3	Alive
26–28	4000	149	0	0	0	0	0	Dead
26–28	4000	164	129	−35	1	0	2	Alive

26–28	4000	149	0	0	0	0	0	Dead
26–28	4000	159	0	0	0	0	0	Dead
26–28	4000	142	141	−1	0	0	0	Alive
26–28	4000	176	0	0	0	0	0	Dead
26–28	4000	158	143	−18	0	0	4	Alive
26–28	4000	173	0	0	0	0	0	Dead
26–28	4000	151	136	−20	0	0	1	Alive
26–28	4000	167	0	0	0	0	0	Dead
26–28	4000	145	0	0	0	0	0	Dead
26–28	4000	182	167	−16	3	0	5	Alive
26–28	4000	154	0	0	0	0	0	Dead
26–28	4000	168	0	0	0	0	0	Dead
26–28	4000	179	164	−23	1	1	3	Alive

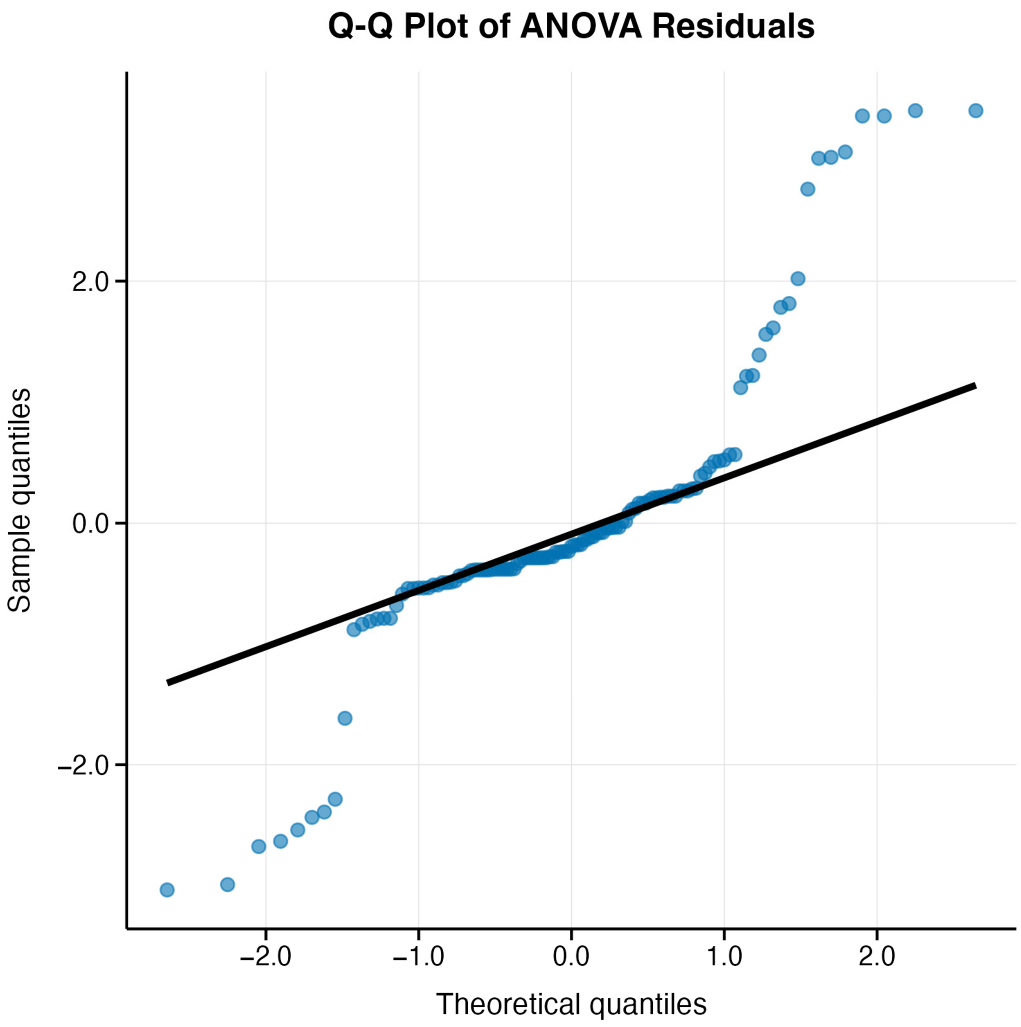

Figure S1. Q–Q plot of ANOVA residuals for body weight changes; blue dots—empirical quantiles against theoretical normal distribution; black diagonal line—ideal normal approximation; Shapiro–Wilk test $W = 0.85$, $p < .001$ indicates deviation from normality; Levene test confirmed homogeneity of variances ($p = .35$); $n = 123$ surviving larvae.
